# The Novel Synthetic Peptide AESIS-1 Exerts a Preventive Effect on Collagen-Induced Arthritis Mouse Model via STAT3 Suppression

**DOI:** 10.3390/ijms21020378

**Published:** 2020-01-07

**Authors:** Kyung Eun Kim, Suwon Jeon, Jisun Song, Tae Sung Kim, Min Kyung Jung, Myun Soo Kim, Sunyoung Park, Seung Beom Park, Jeong Min Park, Hyun Jeong Park, Daeho Cho

**Affiliations:** 1 Department of Cosmetic Sciences, Sookmyung Women’s University, Chungpa-Dong 2-Ka, Yongsan-ku, Seoul 04310, Korea; kyungeun@sookmyung.ac.kr; 2 Nano-Bio Resources Center, Sookmyung Women’s University, Chungpa-Dong 2-Ka, Yongsan-ku, Seoul 04310, Korea; alsrud890@hanmail.net; 3 Institute of Convergence Science, Korea University, Anam-ro 145, Seongbuk-ku, Seoul 02841, Korea; suwongo1@naver.com; 4 Department of Life Sciences, College of Life Sciences and Biotechnology, Korea University, Anam-dong 5-ga, Seongbuk-gu, Seoul 02841, Korea; wltjs529@korea.ac.kr (J.S.); tskim@korea.ac.kr (T.S.K.); 5 Kine Sciences, 525, Seolleung-ro, Gangnam-gu, Seoul 06149, Korea; blackfeather@korea.ac.kr (M.S.K.); sunyoung1101@hanmail.net (S.P.); 6 Cent’l Res. Inst., Ilyang Pharm. Co., Ltd., Hagal-ro 136beon-gil, Giheung-gu, Yongin-si, Gyeonggi-do 17096, Korea; sbpark@ilyang.co.kr (S.B.P.); parkjeongmin@ilyang.co.kr (J.M.P.); 7 Department of Dermatology, Yeouido St. Mary’s Hospital, The Catholic University of Korea, Seoul 07345, Korea

**Keywords:** rheumatoid arthritis, collagen-induced arthritis, peptide, SOCS3, STAT3, Th17 cells

## Abstract

Rheumatoid arthritis (RA) is a chronic autoimmune disease that is associated with systemic inflammation and results in the destruction of joints and cartilage. The pathogenesis of RA involves a complex inflammatory process resulting from the action of various proinflammatory cytokines and, therefore, many novel therapeutic agents to block cytokines or cytokine-mediated signaling have been developed. Here, we tested the preventive effects of a small peptide, AESIS-1, in a mouse model of collagen-induced arthritis (CIA) with the aim of identifying a novel safe and effective biological for treating RA. This novel peptide significantly suppressed the induction and development of CIA, resulting in the suppression of synovial inflammation and cartilage degradation in vivo. Moreover, AESIS-1 regulated JAK/STAT3-mediated gene expression in vitro. In particular, the gene with the most significant change in expression was *suppressor of cytokine signaling 3* (*Socs3*), which was enhanced 8-fold. Expression of the STAT3-specific inhibitor, *Socs3*, was obviously enhanced dose-dependently by AESIS-1 at both the mRNA and protein levels, resulting in a significant reduction of STAT3 phosphorylation in splenocytes from severe CIA mice. This indicated that AESIS-1 regulated STAT3 activity by upregulation of SOCS3 expression. Furthermore, IL-17 expression and the frequency of Th17 cells were considerably decreased by AESIS-1 in vivo and in vitro. Collectively, our data suggest that the novel synthetic peptide AESIS-1 could be an effective therapeutic for treating RA via the downregulation of STAT3 signaling.

## 1. Introduction

Rheumatoid arthritis (RA) is a chronic autoimmune disease characterized by synovial inflammation, cartilage degradation, and joint destruction [1]. Although the pathological mechanisms of RA are not fully understood, both autoimmune and systemic inflammatory responses appear closely associated with the development and progression of RA. Various immune cells, including T cells, B cells, and macrophages, as well as soluble factors such as inflammatory cytokines, autoantibodies, and immune complexes are involved in RA pathogenesis [2]. This disease is initiated by dendritic cell–T cell interactions in peripheral lymphoid organs, leading to B-cell activation resulting from proinflammatory cytokine production from T cells, such as interleukin (IL)-4, IL-10, and IL-13. Activated B cells, in turn, produce numerous autoantibodies that form immune complexes that are deposited in joints, where they induce inflammatory responses in the synovium. In the synovium, the immune complexes activate mast cells, macrophages, and neutrophils to release various proinflammatory cytokines, including tumor necrosis factor (TNF), IL-6, vascular endothelial growth factor (VEGF), and chemokines, such as C–C motif ligand (CCL)2 and CCL3, leading to significantly increased immune cell infiltration and synovial pannus formation [2]. The increased levels of proinflammatory cytokines and chemokines enhance the activation of synovial fibroblasts, which migrate into unaffected regions of the synovium of patients with RA. This process, combined with the proliferation of synovial fibroblasts, promotes systemic inflammatory responses [2,3]. Moreover, activated synovial fibroblasts, osteoclasts, and infiltrated immune cells release reactive oxygen species (ROS), nitric oxide (NO), and proteases such as matrix metalloproteases (MMP), as well as proinflammatory cytokines that accelerate the inflammatory responses, resulting in bone destruction and cartilage damage [2,4].

Due to the importance of proinflammatory cytokines and inflammation-related signaling in RA pathogenesis, numerous therapeutic agents targeting these proteins are under development. Current RA therapies often involve biologicals, including neutralizing antibodies or inhibitors, and the therapeutic effects of some agents have been significant [5,6]. Nevertheless, biologicals are expensive and have been associated with numerous safety concerns. Biologicals such as neutralizing antibodies that comprise protein polymers can cause drug resistance, infections, or autoimmune reactions if not completely degraded in the body [7,8]. Several adverse effects resulting from Janus kinase (JAK) inhibitors, such as the development of opportunistic and viral infections, have also been reported [9,10,11]. To overcome these side effects, peptide drugs have been the subject of significantly increased research and development in recent years [5,6,12]. The advantages of peptide drugs include good efficiency and safety profiles, high selectivity, high potency, low tissue accumulation, and low cost [6]. Thus, safe and effective novel peptide therapeutics should be developed to treat RA.

The Janus kinase/signal transduction and activator of transcription (JAK/STAT) signaling pathway is activated by proinflammatory cytokines and induces various processes that promote chronic inflammatory diseases, such as RA, as well as tumorigenesis [13,14]. Levels of proinflammatory cytokines, such as IL-6, and STAT3 activation are significantly increased in patients with RA, and the levels of these proinflammatory cytokines positively correlate with constitutive STAT3 activation [15,16]. Interleukin-6-stimulated STAT3 activation induces the expression of target genes associated with cellular proliferation, the inhibition of apoptosis, and inflammation [17,18]. Specifically, IL-6 plays a critical role in RA pathogenesis by contributing to the activation of various effector cells, including T cells and B cells, leading to Th17 cell differentiation, autoantibody production, and osteoclast formation [8,11,12,19]. Thus, the JAK/STAT signaling pathway plays a critical role in the RA pathogenesis, indicating that the suppression of JAK/STAT pathway could be an effective therapeutic target for RA.

Here, we developed a novel, 19 amino acid peptide, AESIS-1, and investigated its effects and mechanisms of action from the viewpoint of its being a potential agent for treating RA. We found that AESIS-1 exerted significant preventive effects against collagen-induced arthritis (CIA) in a mouse model via downregulating STAT3 activation. Collectively, our data suggest that AESIS-1 represents a promising, effective agent with which to treat RA by downregulating STAT3 signaling.

## 2. Results

### 2.1. Novel Synthetic Peptide AESIS-1 Exerted Preventive Effects on Collagen-Induced Arthritis In Vivo

**Figure 1:**
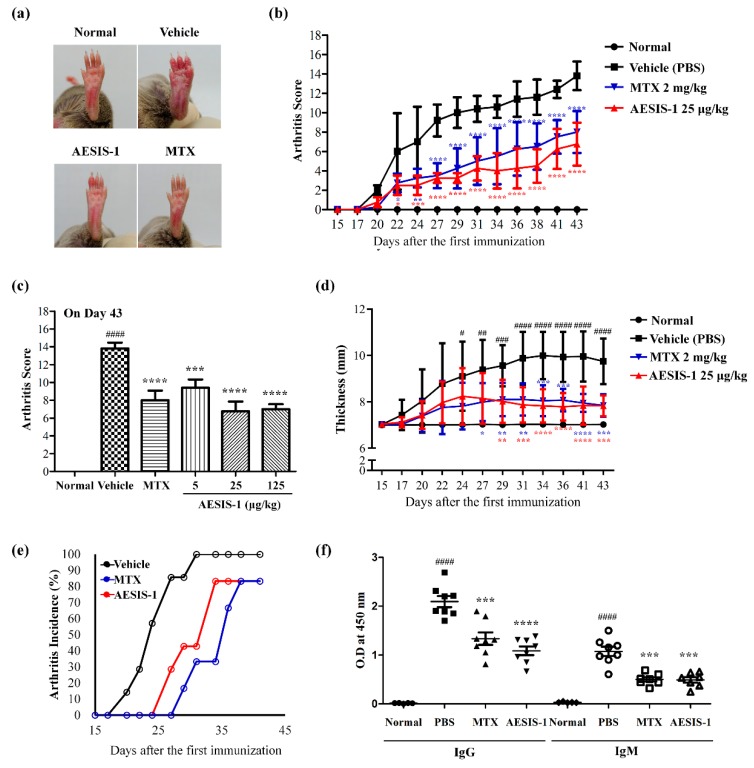
The novel synthetic peptide AESIS-1 exerted preventive effects on collagen-induced arthritis in DBA/1J mice in vivo. Collagen-induced arthritis (CIA) was generated in DBA/1J mice via the subcutaneous (s.c.) injection of type II collagen (CII, 50 μg) into the tail vein, as described in Materials and Methods. One group of CIA mice (*n* = 8) was treated with 25 μg/kg AESIS-1 three times a week by intraperitoneal (i.p.) injection, beginning 1 day after CII boost. Another group was treated with methotrexate (MTX) as a positive control, and vehicle control mice were treated with phosphate-buffered saline (PBS) (*n* = 8 for both groups). (**a**) Gross observation of the hind paw; photographs are of representatives from each group on Day 34; (**b**) Mean arthritic score for each group. The severity was evaluated on a scale from 0 to 4; (**c**) Dose titration of AESIS-1 (0, 5, 25, 125 μg/kg) on Day 43; (**d**) Paw thickness was measured using a dial indicator thickness gauge by three researchers independently; (**e**) Arthritis incidence in each group; (**f**) On Day 28 after the first CII administration, collagen-specific antibodies in the mouse sera were measured using the enzyme-linked immunosorbent assays (ELISA) method after dilution (1:25,000 for IgG and 1:12,500 for IgM). Analysis of variance (ANOVA) with Tukey’s post hoc tests was used for statistical analysis. * *p* ≤ 0.05, ** *p* ≤ 0.01, *** *p* ≤ 0.001, and **** *p* ≤ 0.0001, compared with vehicle (phosphate-buffered saline, PBS) group. ^#^ *p* ≤ 0.05, ^##^ *p* ≤ 0.01, ^###^ *p* ≤ 0.001, and ^####^ *p* ≤ 0.0001, compared with normal group.

To develop a novel synthetic peptide for treating rheumatoid arthritis (RA), we synthesized the novel small molecule peptide AESIS-1 (C_82_H_141_N_31_O_31_S_2_) which consists of 19 amino acids (MSLPSPRDGRTDGRTDCTR) and has a molecular weight (MW) of 2121.4. To investigate the effects of the synthetic peptide on RA, DBA/1J mice were immunized, then subsequently boosted with bovine type II collagen (CII) to promote the development of collagen-induced arthritis (CIA), as described previously [20]. AESIS-1 was administered intraperitoneally (i.p.) to CIA mice three times weekly, beginning on the day after receiving the CII boost. Control mice were treated at the same time points with phosphate-buffered saline (PBS; vehicle control). Figure 1a shows that the CIA mice treated with vehicle displayed severe inflammatory responses, including redness and swelling in the paw, compared with normal mice. Conversely, inflammatory responses in the paw were significantly reduced in CIA mice treated with 25 μg/kg of AESIS-1 compared with those treated with vehicle control. The onset of arthritis was evident around Day 20 in both groups, and mean arthritis scores reached 13.8 and 6.75 in the groups treated with vehicle and AESIS-1 on Day 43, respectively (Figure 1b). Arthritis scores sharply increased in CIA mice treated with vehicle, but AESIS-1 significantly decreased arthritis scores. AESIS-1 dose-dependently prevented these effects in CIA mice, and the most effective dose was 25 μg/kg (Figure 1c). As shown in Figure 1d, paw thickness was also significantly increased and paws in the vehicle group were the most swollen on Day 34 (mean 9.75 mm), indicating inflammation. Severe joint deformations in all toes and severe swelling and redness in the paws were shown in the vehicle group on Day 34 (Figure 1a). We further found that the incidence of arthritis was obviously decreased among CIA mice treated with AESIS-1 compared with those treated with vehicle (Figure 1e). All the mice slowly gained weight, with no significant differences between vehicle and AESIS-1 groups (Figure S1). All mice in all groups survived during the experimental period, implying that AESIS-1 was not toxic in this mouse model.

Autoantibody production is a marker of propagation stage for RA pathogenesis. We therefore measured collagen-specific immunoglobulins, including total IgG and IgM, in the mouse sera using enzyme-linked immunosorbent assays (ELISA). Figure 1f shows that AESIS-1 significantly reduced serum levels of collagen-specific immunoglobulins, indicating that autoantibody production was inhibited. Collectively, these data suggest that the novel synthetic peptide AESIS-1 exerted preventive effects in a mouse model of CIA in vivo.

### 2.2. AESIS-1 Suppressed Synovial Inflammation and Cartilage Degradation In Vivo

**Figure 2:**
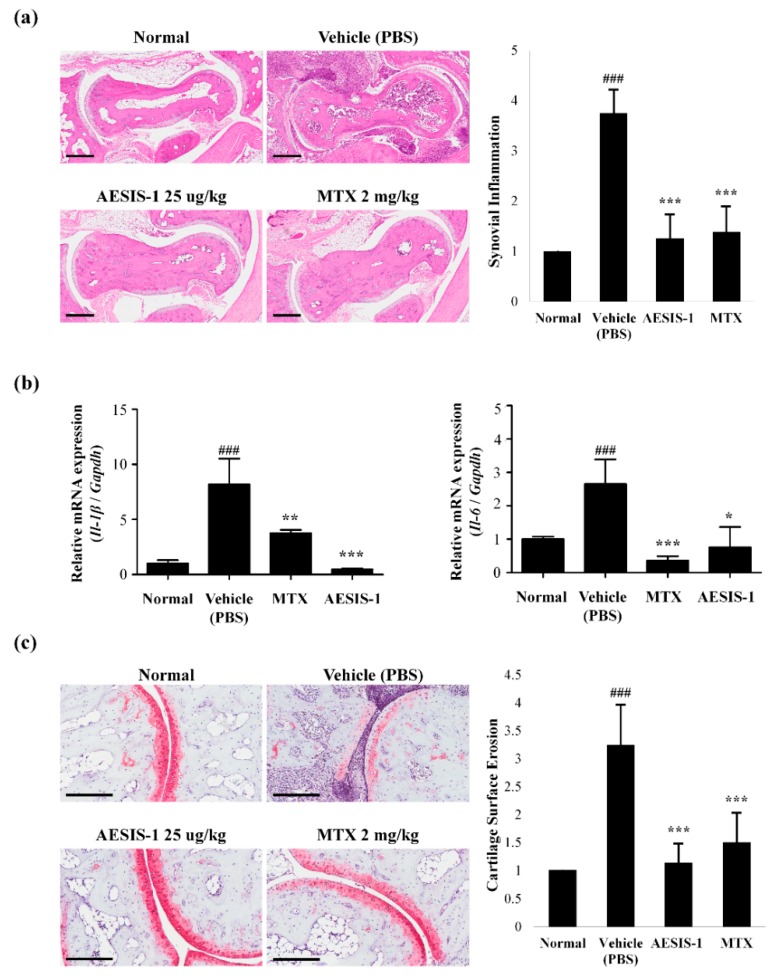
AESIS-1 suppressed synovial inflammation and cartilage destruction in vivo. (**a**) Histological analysis of 8 μm sections from paraffin-embedded hindlimb tissue stained with hematoxylin and eosin. Photographs are of representatives from each group (scale bar, 300 μm). Degree of synovial inflammation was evaluated on a scale from 0 to 4; (**b**) The mRNA expression of proinflammatory cytokines *Il-1β* and *Il-6* was determined by real-time PCR of total RNA isolated from the tissues. The relative mRNA expression level was set to 1 for the normal control; (**c**) For examination of cartilage degradation in synovial tissues, sections were stained with safranin O. Photographs are of representatives from each group (Scale bar, 60 μm). Degree of cartilage surface erosion was also evaluated on a scale from 0 to 4. ANOVA with Tukey’s post hoc tests was used for statistical analysis. ^###^ *p* ≤ 0.001, compared with normal group. * *p* ≤ 0.05, ** *p* ≤ 0.01, and *** *p* ≤ 0.001, compared with vehicle (PBS) group.

We histologically analyzed joint tissues from mice in all groups. CIA mice treated with vehicle (PBS) displayed severe inflammation in the paw joint compared with normal mice. The degree of synovial inflammation on sections stained with hematoxylin and eosin was scored from 0 to 4, as described previously [21]. Figure 2a shows that AESIS-1 considerably reduced synovial inflammation, and that the articular capsules resembled those of normal control mice. In addition, we assessed the mRNA expression of the inflammatory cytokines IL-1β and IL-6 in tissue lysates. Figure 2b shows that AESIS-1 decreased *Il-1β* and *Il-6* mRNA expression, indicating that it has anti-inflammatory effects. In addition to synovial inflammation, safranin O staining revealed that AESIS-1 obviously suppressed cartilage degradation (Figure 2c). All sections were scored in terms of the degree of cartilage surface erosion [21]. Safranin O staining was significantly decreased in joint sections from vehicle-treated CIA mice, indicating proteoglycan depletion and cartilage damage. However, AESIS-1 effectively blocked cartilage degradation of the joint, suggesting that AESIS-1 attenuated RA progression and the degree of tissue damage during RA pathogenesis.

### 2.3. AESIS-1 Significantly Upregulated Negative Regulator of STAT3 Signaling (SOCS3), Resulting in Decreased STAT3 Phosphorylation

**Figure 3:**
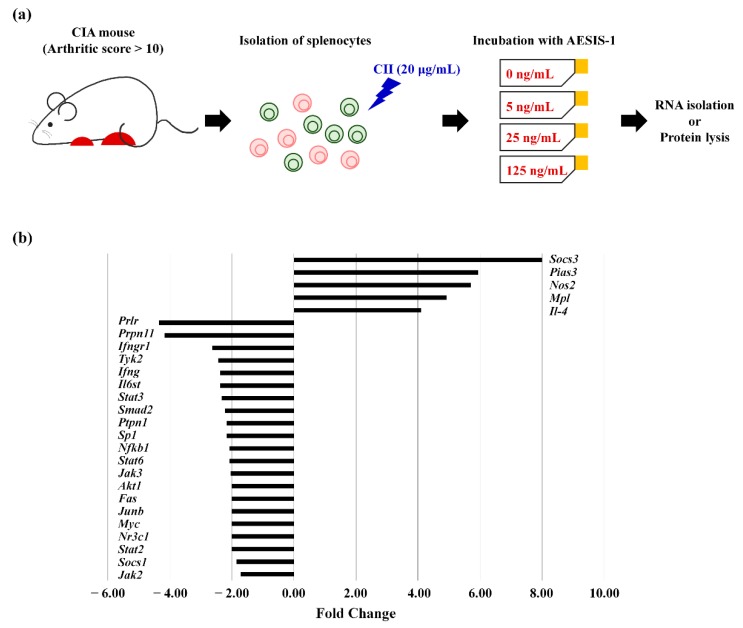

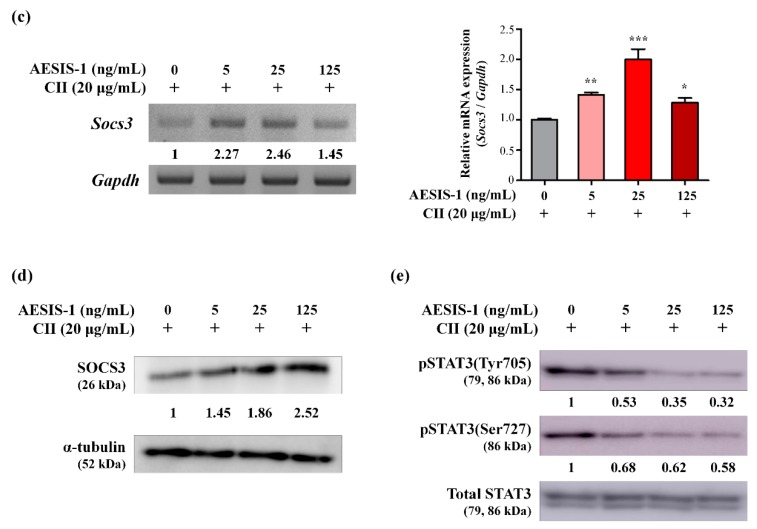
AESIS-1 directly increased SOCS3 expression, resulting in decreased STAT3 phosphorylation. (**a**) Scheme of in vitro experiments. Splenocytes were isolated from CIA mice with an arthritic score >10, and the isolated splenocytes were then incubated with or without AESIS-1 in the presence of 20 μg/mL CII. After incubation for 24 h, total RNA or protein were isolated from the cells; (**b**) To detect JAK/STAT-signaling-related gene expression, total RNA was extracted from CII-treated (20 μg/mL) splenocytes from CIA mice that were incubated with or without AESIS-1 (25 ng/mL) for 24 h. The RT^2^ Profiler PCR Array containing primers for 84 genes related to JAK/STAT signaling was then used to compare the gene expression levels in negative control cells vs. AESIS-1-treated cells; (**c**) To further confirm the effect of AESIS-1 on *Socs3* gene expression, reverse-transcription PCR (RT-PCR) and real-time RT-PCR (qRT-PCR) were performed. The relative mRNA expression level was set to 1 for control cells without AESIS-1. ANOVA with Tukey’s post hoc tests was used for statistical analysis. * *p* ≤ 0.05, ** *p* ≤ 0.01, and *** *p* ≤ 0.001, compared with control cells without AESIS-1; (**d**) The effect of AESIS-1 on SOCS3 protein expression was confirmed by western blot analysis. Western blot analysis with rabbit anti-mouse SOCS3 and α-tubulin antibodies was performed to detect the levels of each protein; (**e**) The effect of AESIS-1 on STAT3 phosphorylation was confirmed by western blot analysis using rabbit anti-mouse phospho-STAT3 (Tyr705), rabbit anti-mouse phospho-STAT3 (Ser727), and rabbit anti-mouse total STAT3 antibodies. Full-length blots of the cropped image as shown in Figure 3d,e are presented in Figure S2.

Helper T(Th)17 cells are involved in the pathogenesis of various autoimmune diseases including RA and psoriasis [22,23]. Interleukin-17 produced by Th17 cells activates synovial fibroblasts, endothelial cells, and infiltrated immune cells in the synovium, leading to promotion of osteoclastogenesis and synovial inflammation during RA pathogenesis [22,24]. STAT3 is a crucial signaling molecule for Th17 cell differentiation [25]; therefore, we examined the effects of AESIS-1 on JAK/STAT-related gene expression in splenocytes, including various subsets of T cells. The splenocytes were isolated from CIA mice with arthritic scores >10, and then incubated with or without AESIS-1 (25 ng/mL) in the presence of 20 μg/mL CII for 24 h (Figure 3a). We then analyzed the expression of 84 genes associated with JAK/STAT signaling using the RT^2^ Profiler PCR Array. Table 1 and Figure 3b show the genes that were up- or downregulated >2-fold by AESIS-1, compared with the control group without AESIS-1. Of these 5 upregulated and 21 downregulated genes, *Socs3* was the most significantly altered by AESIS-1, being increased >8-fold.

We further confirmed the effects of AESIS-1 on *SOCS3* mRNA and protein expression using RT-PCR, real-time PCR, and western blots. Splenocytes isolated from severe CIA mice were incubated without or with 5, 25, and 125 ng/mL of AESIS-1 in the presence of 20 μg/mL CII for 24 h, as described in Figure 3a. Figure 3c shows that AESIS-1 significantly increased *Socs3* mRNA expression compared with control cells without AESIS-1. The increase in expression was the most obvious in cells incubated with 25 ng/mL of AESIS-1. Similar to the mRNA expression, AESIS-1 also dose-dependently increased SOCS3 protein expression (Figure 3d). SOCS3 belongs to the SOCS family, which includes SOCS1-7 and cytokine-inducible SH2-containing protein (CISH), which bind to JAK and cytokine receptors. Because most SOCS3-binding receptors bind STAT3, SOCS3 specifically inhibits STAT3 and consequently suppresses STAT3-mediated signaling [26]. Thus, these data suggested that AESIS-1 could suppress STAT3-mediated signaling via the upregulation of SOCS3 expression.

Because SOCS3 is an established inhibitor of STAT3-mediated signaling, we determined whether AESIS-1 suppresses STAT3 signaling by measuring phospho-STAT3 expression using western blots. Cell lysates were collected as described in Figure 3a. Figure 3e shows that AESIS-1 dose-dependently decreased STAT3 phosphorylation on both Tyr705 and Ser727 (Figure 3e and Figure S2). Collectively, these results confirm that AESIS-1 enhanced SOCS3 expression, resulting in suppressed STAT3 phosphorylation.

### 2.4. AESIS-1 Suppressed IL-17 Production and Th17 Cell Populations

**Figure 4:**
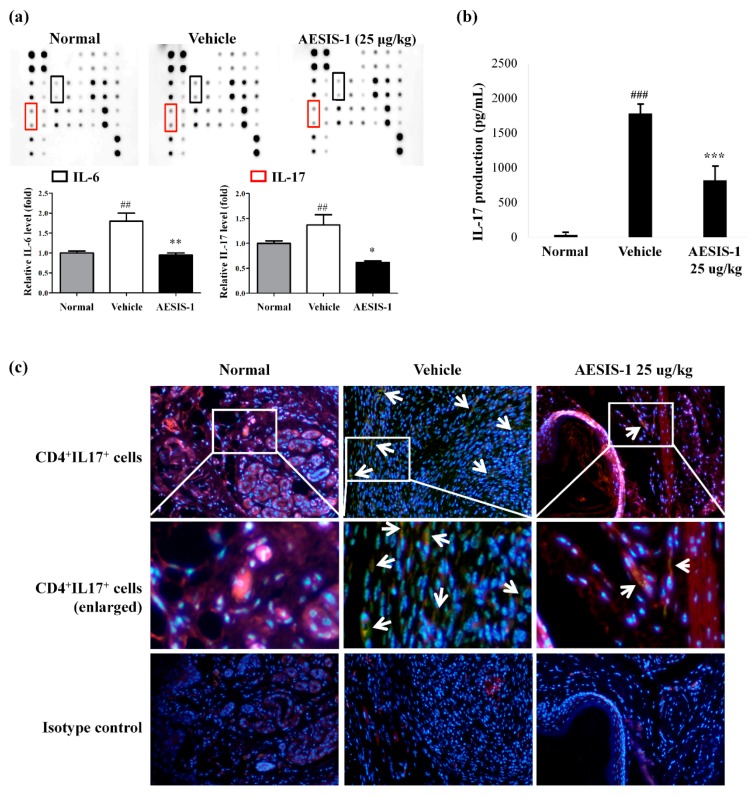
AESIS-1 suppressed IL-17 production and Th17 cell distribution in CIA mouse model. (**a**) To detect IL-6 and IL-17 levels, a cytokine array was performed using the mouse sera collected from normal, vehicle, and AESIS-1 groups. The intensity of each spot was then quantified using ImageJ software. The relative protein level was set to 1 for normal control; (**b**) To measure the levels of IL-17 production, splenocytes were isolated from vehicle- or AESIS-1-treated CIA mice. The cells were stimulated with CII (20 μg/mL) for 24 h, and then ELISA was performed to detect IL-17 levels in the cultured supernatant. ANOVA with Tukey’s post hoc tests was used for statistical analysis. ^##^ *p* ≤ 0.01 and ^###^ *p* ≤ 0.001, compared with normal control. * *p* ≤ 0.05, ** *p* ≤ 0.01, and *** *p* ≤ 0.001, compared with vehicle (PBS) group; (**c**) To detect CD4^+^IL-17^+^ Th17 cell distribution in the tissues, immunofluorescence staining was performed using rat anti-mouse CD4 Ab and rabbit anti-IL-17 Ab as primary antibodies. Alexa-594-conjugated Rat IgG and FITC-conjugated Rabbit IgG antibodies were used then for CD4 and IL-17, respectively. Nuclei were stained by Heochst33258. Rabbit IgG or rat IgG were used as isotype controls. White arrows indicate double-stained cells. Photographs are of representatives of each group (original magnification ×200).

The activation of STAT3 plays a critical role in Th17 cell differentiation, and IL-6 stimulates STAT 3 activation [21]. Specifically, IL-6 plays a critical role in RA pathogenesis by contributing to Th17 cell differentiation. Therefore, IL-6 and IL-17 levels in the mouse sera from normal, vehicle-treated, and AESIS-1-treated groups were measured using a cytokine array. Figure 4a shows that AESIS-1 significantly reduced the increases in both proinflammatory cytokines in sera from vehicle-treated mice. The relative levels of IL-6 and IL-17 were increased in the vehicle-treated CIA mice compared with the normal mice; however, the increased levels were significantly decreased by AESIS-1. Next, we examined the amount of IL-17 produced by splenocytes and the distribution of Th17 cells in affected tissues from vehicle- or AESIS-1-treated CIA mice. The data showed obviously increased IL-17 production in supernatants of splenocytes cultured from vehicle-treated CIA mice ex vivo, and significantly reduced production in those from CIA mice treated with AESIS-1 (Figure 4b). Consistent with these findings, the distribution of CD4^+^IL-17^+^ Th17 cells was also decreased in affected tissues from CIA mice treated with AESIS-1 compared with those treated with vehicle (Figure 4c).

**Figure 5:**
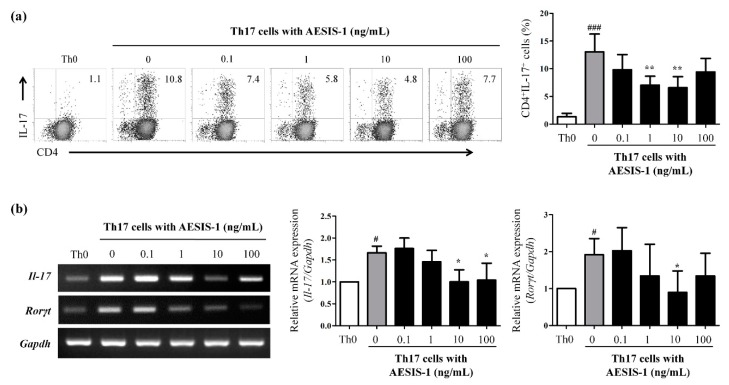
AESIS-1 suppressed the frequency and mRNA expression of Th17 cells in vitro. Naïve CD4^+^ T cells were isolated and purified from lymph nodes of 6 week old DBA/1J mice using magnetic beads. The purified naïve CD4^+^ T cells were cultured with Th17 polarizing cytokines TGF-β (5 ng/mL) and IL-6 (20 ng/mL) in the presence of plate-bound anti-CD3 Ab (1 μg/mL) and soluble anti-CD28 Ab (1 μg/mL) for 3 days. The cells were then incubated without or with AESIS-1 (0.1, 1, 10, 100 ng/mL) for 24 h. (**a**) The proportion of CD4^+^IL-17^+^ Th17 cells was analyzed by flow cytometry as described in Materials and Methods. Results are shown representative of five independent experiments, and the graph representatively show the percentage of CD4^+^IL-17^+^ Th17 cells. All data were shown in Figure S3; (**b**) The mRNA expression of Il-17A and RORγt were analyzed by RT-PCR. Results are shown representative of three independent experiments. ANOVA with Tukey’s post hoc tests was used for statistical analysis. ^#^ *p* ≤ 0.05 and ^###^ *p* ≤ 0.001, compared with Th0 cells. * *p* ≤ 0.05 and ** *p* ≤ 0.01, compared with Th17 cells without AESIS-1.

To further identify the direct effects of AESIS-1 on Th17 cells, lymphocytes were isolated from lymph nodes of DBA/1J mice, and naïve CD4^+^ T cells were then purified from the isolated lymphocytes using magnetic beads, as described in Materials and Methods. The purified naïve CD4^+^ T cells were cultured with the Th17 polarizing cytokines TGF-β and IL-6 in the presence of anti-CD3 and anti-CD28 antibodies. After incubation for 3 days, the cells were incubated without or with 0.1, 1, 10, 100 ng/mL AESIS-1 to determine the direct effects of AESIS-1 on the frequency of Th17 cells. As shown in Figure S3, under the Th17-polarizing conditions, the percentage of CD4^+^IL-17^+^ double positive cells was increased to about 10–17%. However, AESIS-1 dose-dependently reduced the frequency of polarized CD4^+^IL-17^+^ cells, and the most effective dose was 10 ng/mL (Figure 5a). The number of CD4^+^IL-17^+^ cells and the mRNA expressions of *Il-17A* and *RORγt* were decreased in the group given AESIS-1 (Figure 5). Overall, these results show that AESIS-1 directly affected the number of certain Th17 cell populations, supporting the notion that AESIS-1 could serve as a treatment for RA.

## 3. Discussion

Rheumatoid arthritis (RA) is an autoimmune disease associated with chronic inflammation. Many studies have found that both autoimmune and systemic inflammatory responses are closely associated with RA development and progression [2]. Various proinflammatory cytokines produced by immune cells, such as T cells and B cells, are key drivers of the severe systemic inflammatory responses elicited during RA pathogenesis [2]. Thus, inhibiting the production of proinflammatory cytokines and related signaling mediators to treat RA has been a major therapeutic focus over the past three decades.

Here, the novel synthetic peptide AESIS-1 exerted significant beneficial effects that resulted in decreased synovial inflammation and cartilage destruction in a CIA mouse model (Figure 1 and Figure 2). The present study targeted the regulation of JAK/STAT3 signaling to elucidate the mechanisms associated with RA improvement. Figure 3 shows that AESIS-1 regulated various JAK/STAT3-related genes, of which *Socs3* was the most highly upregulated (>8-fold). A previous study found that both arthritis severity and IL-6 production are significantly decreased in CIA mice that overexpress SOCS3 [27]. Additionally, a loss of SOCS3 enhances IL-17 production in CD4^+^ T cells and promotes IL-1-induced inflammatory joint disease [28]. These findings suggest that SOCS3 functions in the regulation of RA-related proinflammatory cytokines, including IL-1, IL-6, and IL-17. This study also showed that AESIS-1, which induced *SOCS3*, obviously suppressed production of the proinflammatory cytokines, IL-1β, IL-6, and IL-17, as well as STAT3 phosphorylation. This resulted in a decrease in IL-17 production by splenocytes and reduced the numbers of Th17 cells in affected tissues. The JAK/STAT3 signaling pathway is stimulated by several proinflammatory factors to induce RA progression and is inhibited by negative regulators, including SOCS3 [29]. Therefore, our data suggest that AESIS-1 ameliorated collagen-induced arthritis by suppressing the STAT3 signaling pathway via significantly upregulating the negative regulator of STAT3. In addition to RA, SOCS3 also plays critical roles in other inflammatory diseases in which STAT3 is activated, including intestinal bowel disease, multiple sclerosis, and allergies [26]. A deletion of SOCS3 in keratinocytes promotes psoriasis-like skin inflammation via STAT3 hyperactivation [30]. Therefore, further studies are needed to determine a potential therapeutic role for AESIS-1 in the upregulation of SOCS3 expression in other autoimmune diseases, such as psoriasis.

Following the European League Against Rheumatism (EULAR) and American College of Rheumatology (ACR) guidelines [31,32], methotrexate (MTX), as the most common disease-modifying anti-rheumatic drug (DMARD), serves as first-line therapy. Most patients with newly diagnosed RA have undergone MTX monotherapy. However, only about 30% of patients with RA develop adequate clinical responses to MTX within 6 months [33]. Patients who do not respond to MTX monotherapy can be treated with combinations of other DMARD and biologicals or with biological monotherapy including anti-tumor necrosis factor (TNF) agents [34]. The most popular biologicals currently in use to treat RA are neutralizing antibodies against proinflammatory cytokines, including tumor necrosis factor inhibitors (such as adalimumab, infliximab, and etanercept), anti-CD20 neutralizing antibodies (rituximab), T-cell co-stimulation inhibitors (abatacept), and IL-6R blockers (tocilizumab) [35,36,37]. In addition to inhibitors of proinflammatory cytokines, those that directly inhibit cytokine-mediated signaling have also shown therapeutic efficacy against RA. Tofacitinib, baricitinib, and decernotinib, which are JAK-specific inhibitors, are clinically effective against RA [38,39,40]. However, they are associated with numerous side effects, including drug resistance and the stimulation of autoimmune responses resulting from their non-biodegradability, and an increased incidence of opportunistic and viral infections [11]. Therefore, many studies are currently underway to develop novel therapies to overcome these limitations of current biologicals.

Here, we examined the preventive effects of a novel synthetic peptide for treating RA compared with conventional treatment including MTX and Enbrel (etanercept; an anti-TNF agent). The CIA mice developed arthritis more slowly when treated with AESIS-1 than MTX. Furthermore, AESIS-1 was much more effective than Enbrel, as shown in Figure S4. Arthritis prevention significantly differed between mice treated with AESIS-1 and the positive controls, i.e., MTX and Enbrel. The preventive effects of AESIS-1 were excellent at 25 μg/kg, compared with MTX at 2 mg/kg and Enbrel at 5 mg/kg. In other words, it was found that AESIS-1 effectively prevents arthritis even at relatively low concentrations. In general, peptide drugs have various advantages, such as low toxicity, high selectivity, high potency, low tissue accumulation, and biological diversity [6]. Here, AESIS-1 effectively prevented the development of RA in CIA mice, without either cytotoxicity in various cell lines in vitro (Figure S5) or toxicity in mice in vivo (Figure S1), suggesting that it could serve as an effective and safe therapeutic. However, further investigations are needed to prove that AESIS-1 can overcome the limitations of conventional therapeutic agents such as nonbiodegradability, drug resistance, and the stimulation of autoimmune responses, as no direct comparative studies are currently available.

Additionally, this study aimed to determine the effects and mechanism of AESIS-1 action in CIA model mice, but gaps in our knowledge remain. Generally, peptides are highly selective signaling molecules which, when bound to specific cell surface receptors such as G-protein-coupled receptors, ion channels, or intracellular receptors, trigger intracellular signals through receptor internalization [5]. Thus, to identify the mode of action of AESIS-1 in RA, future studies should explore binding proteins and receptors for AESIS-1. We also designed a means to determine the preventive effects of the peptide in vivo. Because AESIS-1 conferred significant preventive effects in CIA model mice, we suggest that it has potential as a treatment for RA. Furthermore, AESIS-1 suppressed STAT3 activation in splenocytes isolated from mice with severe CIA and decreased the number of polarized Th17 cells, implying that it could be an effective therapeutic agent for treating various autoimmune diseases, including RA, characterized as STAT3-hyperactivated or Th17-cell-dominant. Thus, future studies are needed to prove the therapeutic effects of AESIS-1 after the onset of arthritis in the CIA model mice.

## 4. Materials and Methods

### 4.1. Novel Synthetic Peptide AESIS-1

The novel small molecule peptide AESIS-1 contains 19 amino acids and has an expected molecular weight of 2121.4 Da (Peptron, Inc., Daejeon, Korea). AESIS-1 peptide was generated using the ASP48S (Peptron, Inc.), using the solid phase peptide synthesis (SPSS) method. High-purity peptide was obtained from the major fraction of the synthesized peptide pool using SHIMADZU Prominence HPLC (Shimadzu, Kyoto, Japan). Purified synthetic peptide was prepared by the freeze-drying method. Purity of synthetic peptide (>95%) was assessed by SHIMADZU Prominence HPLC and peptide molecular weight was determined by liquid chromatography–mass spectrometry (LC-MS) using the SHIMADZU LCMS-2020 (Shimadzu). The properties of AESIS-1 peptide are listed in Table 2. 

### 4.2. Ethics Statement

Mouse experimental procedures were reviewed and approved by the Institutional Animal Care and Use Committee of Sookmyung Women’s University, Republic of Korea (21 Nov 2017; SMWU-IACUC-1711-031). All animal experiments were performed according to the approved guidelines and regulations.

### 4.3. Collagen-Induced Arthritis Mouse Model

Male, 6 week old DBA/1J mice (Central Lab. Animal Inc., Seoul, Korea) were used in a collagen-induced model of arthritis according to published protocols [20]. Bovine type II collagen (CII, 2 mg/mL, Chondrex, Redmond, WA, USA) was diluted in an equal volume of complete Freund’s adjuvant (Chondrex). Mice were then immunized with a 50 μL emulsion (containing 50 μg CII) through subcutaneous (s.c.) injection into the tail vein. After 14 days, a 50 μL booster injection containing a 1:1 emulsion of CII (50 μg) and incomplete Freund’s adjuvant (Chondrex) was administered through s.c. injection into the tail vein. Beginning 1 day after boosting, mice were then treated with AESIS-1 (0, 5, 25, 125 μg/kg) via intraperitoneal (i.p.) injection three times a week; phosphate-buffered saline (PBS) was administrated as a vehicle control. Each experimental group contained eight mice, and all reported data are from three independent experiments.

### 4.4. Evaluation of Arthritic Severity

Arthritic severity was scored individually based on previously published protocols. The severity of paw inflammation was evaluated on a scale of 0 to 4 [20]. Scores were graded by researchers who were blinded to the animal grouping, and the sum of scores from each paw was reported as the arthritic score. In addition, paw thickness was also measured using a dial indicator thickness gauge (Mitutoyo, Kawasaki, Japan) by three researchers to detect the paw inflammation, such as swelling.

### 4.5. Measurement of Collagen-Specific Ig Titers

Blood samples were collected from the tail vein, 28 days after the first immunization, and serum was isolated after clotting at room temperature (RT) for 30 min. To determine collagen-specific immunoglobulins, such as total IgG and IgM, enzyme-linked immunosorbent assays (ELISA) were performed. Briefly, 96 well plates (Nunc, Rochester, NY, USA) were incubated with 4 μg/mL CII in 0.05 M sodium carbonate at 4 °C. After incubation overnight, the wells were washed with Tris-buffered saline containing 0.05% Tween 20 (TBST), and non-specific binding was blocked with 200 μL 1% bovine serum albumin (BSA, Sigma, St. Louis, MO, USA) in TBST at RT for 30 min. Plates were washed again with TBST, and 100 μL of serum diluent (1:25,000 for IgG and 1:12,500 for IgM) was applied and incubated at RT for 2 h. Diluted horseradish peroxidase (HRP)-conjugated goat anti-mouse IgG and IgM (Santa Cruz Biotechnology, Inc., Dallas, TX, USA) was then applied, and plates were incubated at RT for 1 h. After washing again with TBST, 100 μL 3,3′,5,5′-tetramethylbenzidine (TMB, Sigma) was added per well, and samples were incubated in the dark for 20 min, after which 50 μL stop reagent for TMB substrate was added (Sigma), and absorbance was measured at 450 nm.

### 4.6. Evaluation of Synovial Inflammation and Cartilage Degradation

We evaluated synovial inflammation and cartilage degradation in skinned hindlimbs from each mouse. The tissues were fixed in 4% paraformaldehyde (PFA) overnight, decalcified, and then embedded in paraffin blocks. Sections (8 μm thick) were stained with hematoxylin and eosin to visualize the nucleus and cytoplasm. For examination of cartilage degradation in synovial tissues, sections were stained with safranin O. The degree of synovial inflammation and cartilage degradation was scored based on previously published methods [21].

### 4.7. JAK/STAT Gene Expression Array

Splenocytes were isolated from CIA mice with an arthritic score >10. The isolated splenocytes were plated 2 × 10^6^/mL in T25 flasks and incubated in RPMI1640 media (WelGENE Inc., Daegu, Korea) supplemented with 10% heat-inactivated fetal bovine serum (FBS, WelGENE Inc.) in a 5% CO_2_ incubator at 37 °C. The cells were incubated without or with AESIS-1 (25 ng/mL) in the presence of 20 μg/mL CII for 24 h, as described in Figure 3a. To detect JAK/STAT-signaling-related gene expression, total RNA was extracted from cultured splenocytes using TRIzol reagent (Invitrogen, Carlsbad, CA, USA). Equal amounts of RNA were used for synthesis of cDNA using SuperMix Solution (Invitrogen). The RT^2^ Profiler PCR Array, containing primers for 84 genes related to JAK/STAT signaling (QIAGEN, Hilden, Germany), was used to assess gene expression levels. Real-time reverse-transcription PCR (qRT-PCR) was performed with SYBR Green (QIAGEN) according to the manufacture’s instructions, and genes with significantly altered expression are listed in Table 1.

### 4.8. Reverse Transcription Polymerase Chain Reaction (RT-PCR)

Total RNA was extracted, and RT-PCR and qRT-PCR were performed to detect mRNA expression of *Il-1β*, *Il-6*, *Socs3*, *Il-17A*, and *Rorγt*. The specific primer pairs used were as follows; IL-1β sense 5′-GCTTCAAATCTCGCAGCAGC-3′ and antisense 5′-TCACAGAGGATGGGCTCTTC-3′; IL-6 sense 5′-GAAAAGAGTTGTGCAATGGC-3′ and antisense 5′-GTACTCCAGAAGACCAGAGGA-3′; SOCS3 sense 5′-ATGGTCACCCACAGCAAGTTT-3′ and antisense 5′-TCCAGTAGAATCCGCTCTCCT-3′; IL-17A sense 5′-CGCAAAAGTGAGCTCCAGAA-3′ and antisense 5′-TGAAAGTGAAGGGGCAGCTC-3′; RORγt sense 5′-ACCTCCACTGCCAGCTGTGTGC-3′ and antisense 5′-TTTCTGCACTTCTGCATGTAGACTGTCCC-3′; and GAPDH sense 5′-ACATCAAGAAGGTGGTGAAG-3′; antisense 5′-ATTCAAGAGAGTAGGGAGGG-3′. Fold expression for each gene relative to controls was determined after normalization to *Gapdh* expression.

### 4.9. Western Blot Analysis

Splenocytes were isolated from CIA mice with an arthritic score >10, and then the isolated splenocytes were incubated without or with various concentration of AESIS-1 (5, 25, 125 ng/mL) in the presence of 20 μg/mL CII for 24 h, as described in Figure 3a. Total protein was isolated using cell lysis buffer with phosphatase inhibitors (Invitrogen). Equal amounts of each sample (50 μg) were separated on SDS-PAGE gels and transferred to polyvinylidene (PVDF) membranes. These were blocked with 5% non-fat dry milk in PBS at RT for 1 h. After washing, the membranes were incubated overnight at 4 °C with rabbit anti-mouse phospho-STAT3 (Tyr705), phospho-STAT3 (Ser727), total STAT3, SOCS3, or α-tubulin antibodies (Cell Signaling Technology, Danvers, MA, USA). The membranes were then treated with HRP-conjugated goat anti-rabbit IgG (Jackson Immuno Research, West Grove, PA, USA) secondary antibody, and specific bands were visualized with ECL Solution (Westsave, Korea), according to manufacturer’s instructions. The Amersham Imager 600 (GE Healthcare, Chicago, IL, USA) and LAS-3000 chemiluminescence-imaging device (Fujifilm, Tokyo, Japan) were utilized for all data analysis.

### 4.10. Enzyme-Linked Immunosorbent Assay (ELISA)

Splenocytes of normal, vehicle (PBS)-treated, or AESIS-1 (25 μg/kg)-treated CIA mice were isolated. Equal numbers of cells (2 × 10^6^/mL) were incubated with CII (20 μg/mL) in a 5% CO_2_ incubator at 37 °C for 24 h. The cultured supernatants were used to detect IL-17 production. IL-17 ELISA (R&D systems, Minneapolis, MN, USA) was performed according to the manufacturer’s protocols.

### 4.11. Cytokine Array

Blood samples were collected from normal, vehicle (PBS)-treated, and AESIS-1-treated mice that were sacrificed on Day 43, and serum was isolated after clotting at RT for 30 min. We determined circulating levels of various cytokines, including IL-6 and IL-17, in the mouse sera using a cytokine array kit (RayBiotech, Peachtree Corners, GA, USA). Serum samples were diluted 10-fold in dilution buffer, and the array was applied as described by the manufacturers. All data were analyzed using a LAS-3000 chemiluminescence-imaging device (Fujifilm, Tokyo, Japan), and the intensity of each spot was measured using ImageJ software (National Institute of Health, MD, USA).

### 4.12. Immunofluorescence Staining

To detect Th17 cells in the tissues, rabbit anti-IL-17 (Abcam, Cambridge, UK) and rat anti-mouse CD4 (BD Biosciences, San Diego, CA, USA) were used as primary antibodies. Either rabbit IgG (Abcam) or rat IgG2a (BD Biosciences) was used as isotype control. Briefly, 8 μm sections were fixed with cold acetone for 5 min and blocked with 5% goat serum in PBS with 0.1% tritonX-100. The sections were then incubated with primary antibodies at 4 °C. After incubation overnight, the sections were washed with PBS three times, and then incubated with secondary antibodies, FITC-conjugated rabbit IgG (Sigma), and Alexa-594-conjugated Rat IgG (Invitrogen). Nucleus staining with Hoechst 33258 (Invitrogen) was performed as counterstaining. Tissues were visualized using a fluorescence microscope.

### 4.13. Flow Cytometry

To determine the direct effects of AESIS-1 on Th17 cells, lymphocytes were isolated from the lymph nodes of 6 week old DBA/1J mice, and naïve CD4^+^ T cells were then purified from the isolated lymphocytes via magnetic isolation using magnetic beads (MACS; Miltenvi Biotec GmbH, Bergisch Gladbach, Germany). To induce Th17 polarization, naïve CD4^+^ T cells were cultured with Th17-polarizing cytokines TGF-β (5 ng/mL) and IL-6 (20 ng/mL) in the presence of plate-bound anti-CD3 (1 μg/mL) and soluble anti-CD28 (1 μg/mL) for 3 days, as described previously [41,42,43]. Various concentrations of AESIS-1 (0.1, 1, 10, 100 ng/mL) were then directly added to the cells. After incubating for 24 h, the cells were stimulated for 5 h with phorbol myristate acetate (50 ng/mL, Sigma), ionomycin (1 μg/mL, Sigma), and brefeldin A (Golgiplug, 1 μg/mL, BD Biosciences). To determine the frequency of Th17 cells, surface CD4 and intracellular IL-17 were stained as follows [44,45]. The cells were washed with PBS twice, then stained with FITC-conjugated CD4 antibody (BD Biosciences) for 15 min at RT. Subsequently, the cells were fixed using Cytofix/Cytoperm kit (BD Biosciences) for 20 min at 4 °C. The cells were stained with APC-conjugated IL-17 antibody (eBiosciences) for 1 h at 4 °C. Flow cytometry was performed using a FACSCalibur (BD Biosciences), and the data were analyzed using CellQuest (BD Biosciences).

### 4.14. Statistical Analysis

All significance values were calculated using a one-way analysis of variance (ANOVA) with Tukey’s post hoc tests, and all samples passed D’Agostino–Pearson normality tests (alpha = 0.05). Statistical analyses were performed using GraphPad Prism 8 (GraphPad Software, La Jolla, CA, USA). *p* values ≤ 0.05 were considered statistically significant.

## 5. Conclusions

In conclusion, this study provided evidence that the novel synthetic peptide AESIS-1 exerts preventive effects in CIA model mice, indicating that AESIS-1 could help to prevent the onset of RA. Specifically, we demonstrated that AESIS-1 directly acts as a negative regulator of STAT3 signaling via the upregulation of SOCS3 expression, resulting in decreased Th17 populations. Our findings therefore suggest that AESIS-1 could serve as an effective RA treatment via STAT3 downregulation.

## Figures and Tables

**Table 1:** Genes that were upregulated or downregulated by AESIS-1.

Gene Name (Gene Symbol)	Fold Change	Function in JAK/STAT-Mediated Signaling
**Upregulated**		
*Suppressor of cytokine signaling 3* (*Socs3*)	8.00	Negative regulator of IL-6/JAK2/STAT3
*Protein inhibitor of activated STAT 3* (*Pias3*)	5.94	Negative regulator of STAT3
*Nitric oxide synthase 2* (*Nos2*)	6.70	Genes induced by STAT proteins; Inflammatory response
*Myeloproliferative leukemia virus oncogene* (*Mpl*)	4.92	Receptor that binds and activates JAK proteins
*Interleukin-4* (*Il-4*)	4.10	Lymphocyte activation
**Downregulated**		
*Prolactin receptor* (*Prlr*)	0.23	Receptor that binds and activates JAK proteins
*Protein tyrosine phosphatase, non-receptor type 11* (*Ptpn11*)	0.24	Involved in the immune response
*Interferon (alpha and beta) receptor 1* (*Ifngr1*)	0.38	Receptor that binds and activates JAK proteins
*Tyrosine kinase 2* (*Tyk2*)	0.41	One of the JAKs members
*Interferon γ* (*IFNγ*)	0.42	Transcription factor or regulator that interacts with STAT proteins
*Interleukin 6 signal transducer* (*Il-6st*)	0.42	Receptor that binds and activates JAK proteins
*Signal transducer and activator of transcription 3* (*Stat3*)	0.43	STAT family
*MAD homolog 2 (Drosophila)* (*Smad2*)	0.45	Transcription factor or regulator that interacts with STAT proteins
*Protein tyrosine phosphatase, non-receptor type 1* (*Ptpn1*)	0.46	Regulator of JAK/ STAT pathway
*Trans-acting transcription factor 1* (*Sp1*)	0.46	Other transcription factor and regulator
*Nuclear factor of kappa light polypeptide gene enhancer in B-cells 1, p105* (*Nfkb1*)	0.48	Transcription factor or regulator that interacts with STAT proteins
*Signal transducer and activator of transcription 6* (*Stat6*)	0.48	STAT family
*Janus kinase 3* (*Jak3*)	0.49	Janus kinase activity
*Thymoma viral proto-oncogene 1* (*Akt1*)	0.5	Apoptosis
*Fas (TNF receptor superfamily member 6)* (*Fas*)	0.5	Apoptosis
*Jun-B oncogene* (*Junb*)	0.5	Genes induced by STAT3 proteins
*Myelocytomatosis oncogene* (*Myc*)	0.5	Genes induced by STAT3 proteins
*Nuclear receptor subfamily 3, group C, member 1* (*Nr3c1*)	0.5	Receptor that binds and activates JAK proteins
*Signal transducer and activator of transcription 2* (*Stat2*)	0.5	STAT family
*Suppressor of cytokine signaling 1* (*Socs1*)	0.54	Regulator of JAK/STAT pathway
*Janus kinase 2* (*Jak2*)	0.58	Janus kinase activity

**Table 2:** The properties of the AESIS-1 peptide ^1^.

Name	AESIS-1
Sequence	MSLPSPRDGRTDGRTDCTR
Number of Residues	19
Chemical Formula	C_82_H_141_N_31_O_31_S_2_
Molecular Weight	2121.4 g/mol
Isoelectric Point	8.55
Extinction Coefficient	120·M^−1^cm^−1^
Hydrophilicity Analysis	Basic ^2^
Solubility	Water

^1^http://www.biosyn.com/peptidepropertycalculatorlanding.aspx; ^2^ Sequence composition (in percentage): Acidic 15.79; Neutral 47.37; Basic 21.05; Hydrophobic 15.79.

## Supplementary Materials

Supplementary materials can be found at https://www.mdpi.com/1422-0067/21/2/378/s1.
