# Supplementary material for: The Novel Synthetic Peptide AESIS-1 Exerts a Preventive Effect on Collagen-Induced Arthritis Mouse Model via STAT3 Suppression
Source: Int J Mol Sci. 2020 Jan 7;21(2):378. doi: 10.3390/ijms21020378 (PMC7013888; doi:10.3390/ijms21020378)
Supplement: Supplementary file 1 [file ijms-21-00378-s001.pdf]

Supplementary Figure S1

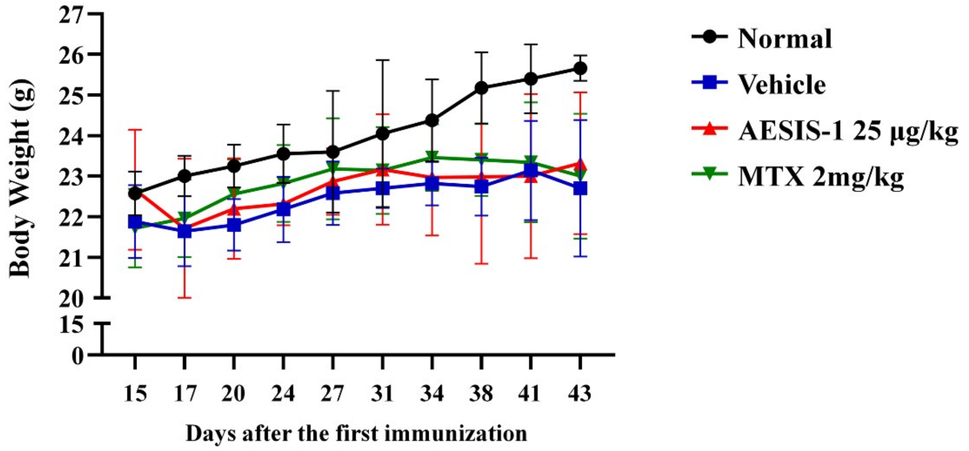

Figure S1. Body weight of CIA mice.

Body weight of normal, vehicle (PBS)-, AESIS-1-, and MTX-treated mice was recorded during 43 days. There was no significant difference between vehicle (PBS)-treated and AESIS-1-treated mice, indicating that AESIS-1 was not toxic in this mouse model.

Supplementary Figure S2

(a)

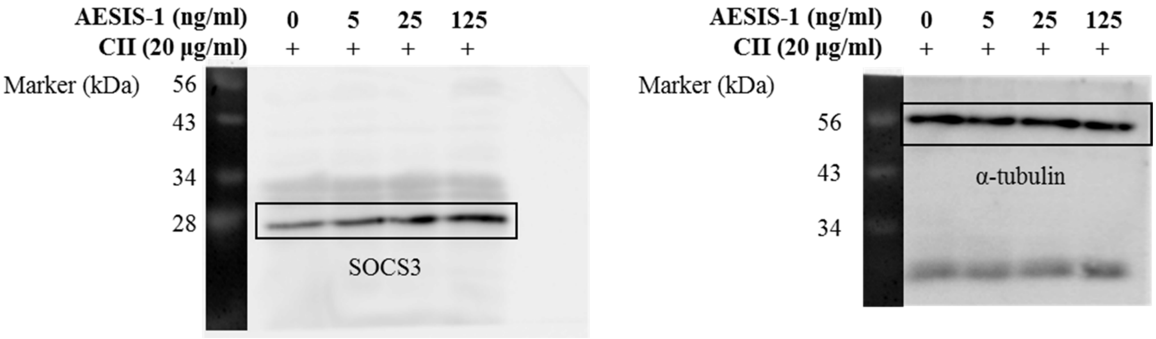

(b)

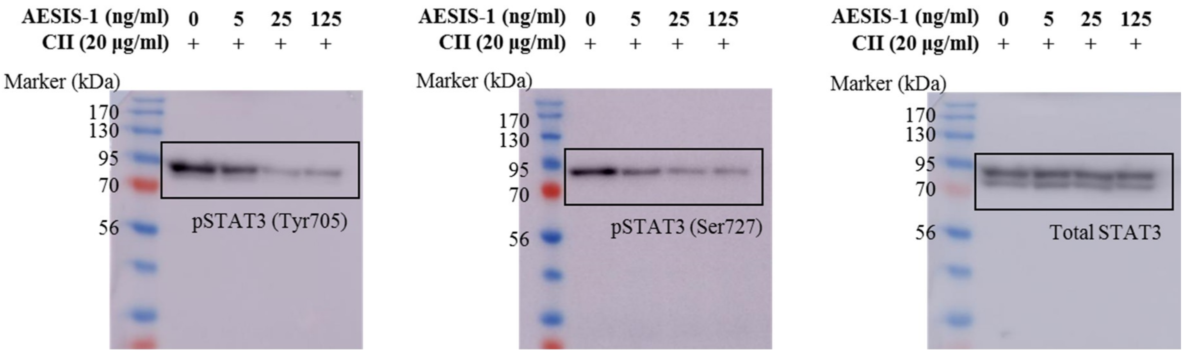

Figure S2. Full-length blots for Figure 3d and 3e.

(a) The indicated square boxes were cropped and used in Figure 3d (SOCS3 and α-tubulin). The specific bands were visualized using ECL solution following the manufacturer's instructions and analyzed using LAS-3000 chemiluminescence-imaging device (Fujifilm). **(b)** The indicated square boxes were cropped and used in Figure 3e (Phosphor-STAT3 (Tyr705), Phosphor-STAT3 (Ser727), and Total STAT3). Total STAT3 staining was performed following stripping the membrane used for pSTAT3 (Tyr705). The specific bands were visualized using ECL solution following the manufacturer's instructions and analyzed using Amersham Imager 600 (GE Healthcare).

Supplementary Figure S3

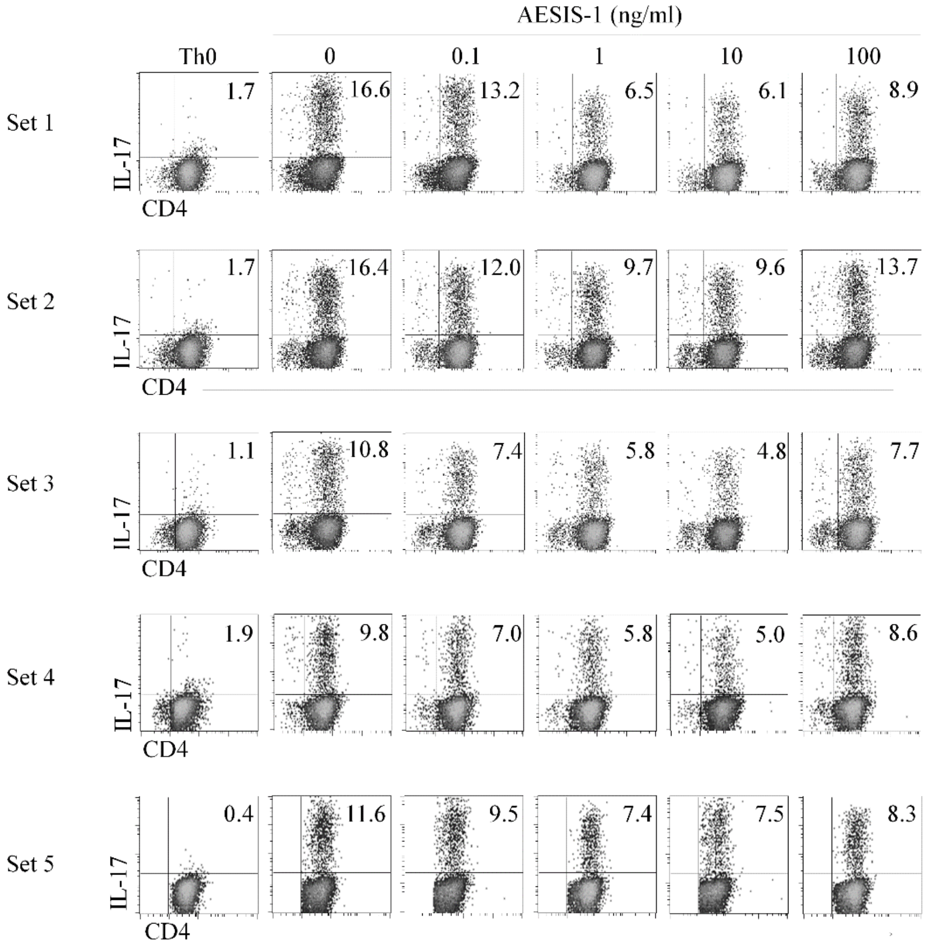

Figure S3. All repeated data for Figure 5a.

Naïve $CD4^+$ T cells were isolated and purified from lymph nodes of 6-weeks old DBA/1J mice using magnetic beads. The purified naïve CD4+ T cells were cultured with Th17 polarizing cytokines, TGF-β (5 ng/mL) and IL-6 (20 ng/mL), in the presence of plate-bound anti-CD3 Ab (1 μg/mL) and soluble anti-CD28 Ab (1 μg/mL) for 3 days. Then, the cells were incubated without or with AESIS-1 (0.1, 1, 10, 100 ng/mL) for 24 h. The proportion of CD4+IL-17+ Th17 cells were analyzed by flow cytometry, and results were shown representative of five independent experiments.

Supplementary Figure S4

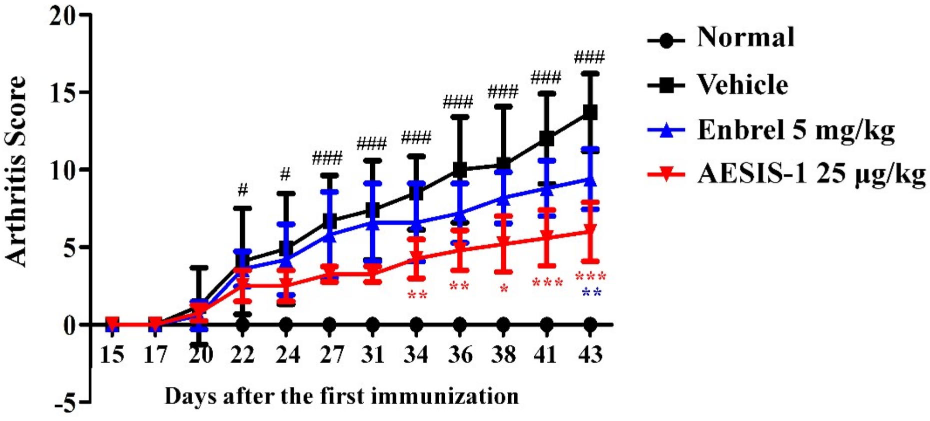

Figure S4. The preventive effects of AESIS-1 on CIA, compared with Enbrel.

Collagen-induced arthritis (CIA) was generated in DBA/1J mice via the subcutaneous (s.c.) injection of type II collagen (CII, 50 μg) into the tail vein as described in Materials and Methods. One group of CIA mice (n = 8) was treated with 25 μg/kg AESIS-1, three times a week, by intraperitoneal (i.p.) injection, beginning one day after CII boost. Another group was treated with Enbrel (5 mg/kg) as a positive control, and vehicle control mice were treated with phosphate-buffered saline (PBS) (n = 8, for both groups). The graph showed a mean arthritic score for each group. ANOVA with Tukey post hoc tests was used for statistical analysis. #$P \leq 0.05$ and ###$P \leq 0.001$, compared with normal group. *$P \leq 0.05$, **$P \leq 0.01$, and ***$P \leq 0.001$, compared with vehicle (PBS) group.

Supplementary Figure S5

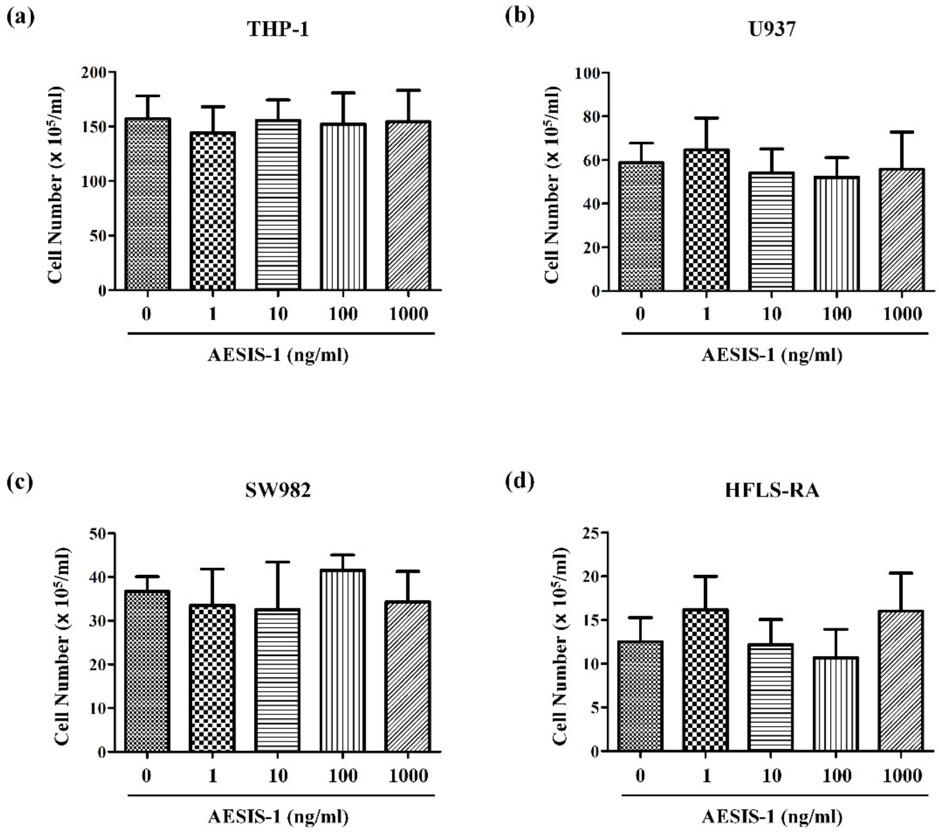

Figure S5. The effect of AESIS-1 on cell growth.

To test the cellular cytotoxicity of AESIS-1, cell number was determined using trypan blue exclusive assay. The cell lines were maintained in RPMI1640 medium (THP-1 and U937), DMEM medium (SW982), or Basal medium (HFLS-RA) containing 10% heat-inactivated FBS in a 5% CO_2 incubator at 37°C. The cells were incubated without or with the indicated concentrations of AESIS-1 for 72 h, and then cell number was counted. (a) THP-1, human monocytic cell line, (b) U937, human monocytic cell line, (c) SW982, human synovial cell line, (d) HFLS-RA, Human Fibroblast-Like Synoviocytes from donor with Rheumatoid Arthritis.
